# Molecular landscape of congenital vertebral malformations: recent discoveries and future directions

**DOI:** 10.1186/s13023-024-03040-0

**Published:** 2024-01-30

**Authors:** Anna Szoszkiewicz, Ewelina Bukowska-Olech, Aleksander Jamsheer

**Affiliations:** 1 Department of Medical Genetics, Poznan University of Medical Sciences, Rokietnicka 8, 60-806 Poznan, Poland; 2 Centers for Medical Genetics GENESIS, Dąbrowskiego 77A, 60-529 Poznan, Poland

**Keywords:** Vertebral defects, Klippel–Feil syndrome, Congenital scoliosis, Spondylocostal dysostosis, Butterfly vertebrae, Hemivertebra, Neural tube defects

## Abstract

Vertebral malformations (VMs) pose a significant global health problem, causing chronic pain and disability. Vertebral defects occur as isolated conditions or within the spectrum of various congenital disorders, such as Klippel–Feil syndrome, congenital scoliosis, spondylocostal dysostosis, sacral agenesis, and neural tube defects. Although both genetic abnormalities and environmental factors can contribute to abnormal vertebral development, our knowledge on molecular mechanisms of numerous VMs is still limited. Furthermore, there is a lack of resource that consolidates the current knowledge in this field. In this pioneering review, we provide a comprehensive analysis of the latest research on the molecular basis of VMs and the association of the VMs-related causative genes with bone developmental signaling pathways. Our study identifies 118 genes linked to VMs, with 98 genes involved in biological pathways crucial for the formation of the vertebral column. Overall, the review summarizes the current knowledge on VM genetics, and provides new insights into potential involvement of biological pathways in VM pathogenesis. We also present an overview of available data regarding the role of epigenetic and environmental factors in VMs. We identify areas where knowledge is lacking, such as precise molecular mechanisms in which specific genes contribute to the development of VMs. Finally, we propose future research avenues that could address knowledge gaps.

## Background

**Fig. 1:**
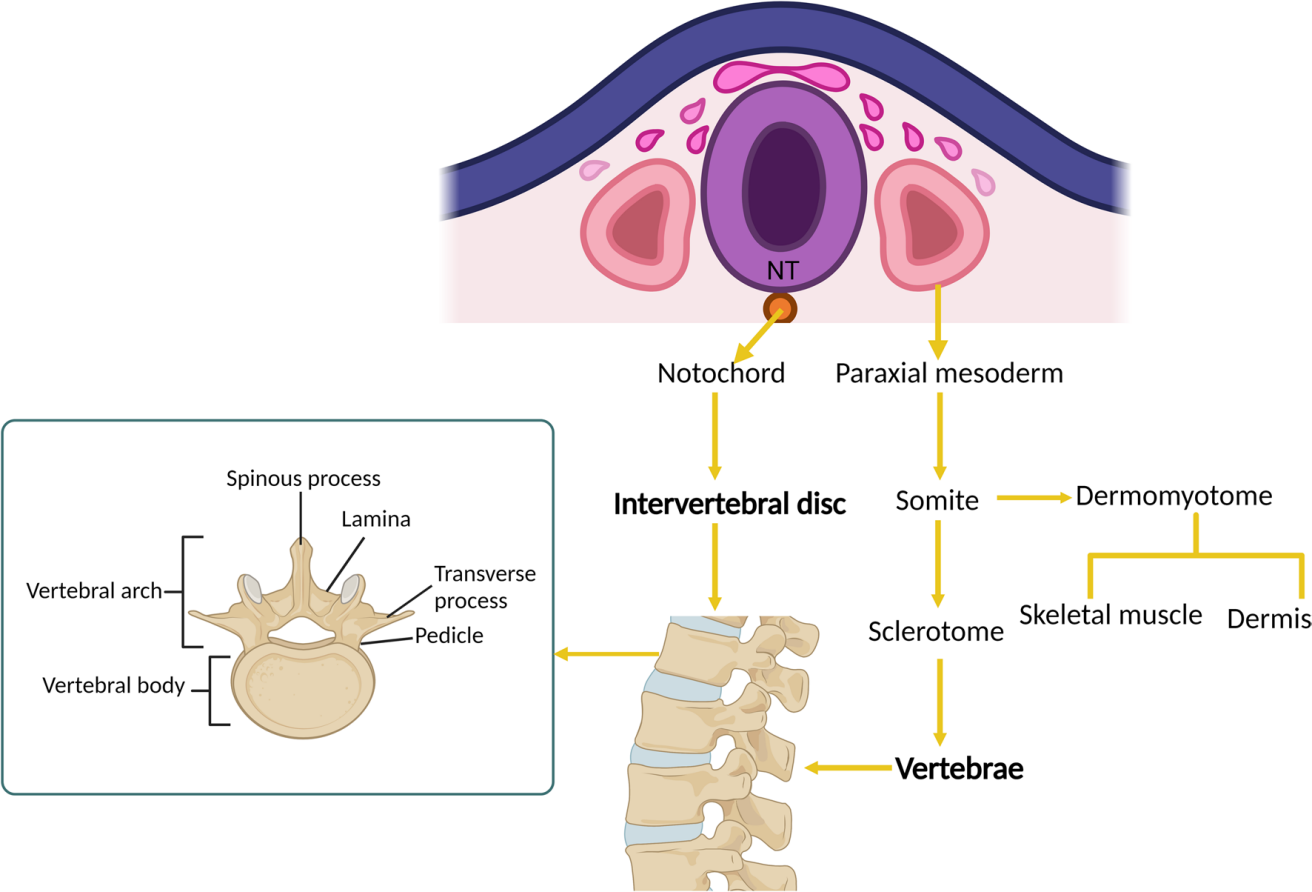
Schematic representation of vertebral development in human embryo. NT – neural tube. Created with Biorender.com

The segmentally organized human vertebral column is built of 31–33 vertebrae, comprising 7 cervical, 12 thoracic, 5 lumbar, 5 sacral, and 2–4 coccygeal vertebrae fused into one bone (i.e. coccyx), housing neurons, the spinal cord, and blood vessels. Development of the embryonic vertebral column is complex, and deep understanding of this process at a molecular level is critical for grasping the origin of vertebral defects. The notochord and somites are the most important structures responsible for the vertebral column formation. Somites develop from the paraxial mesoderm on either side of the midline, and then differentiate into ventromedial sclerotome and dorsolateral dermomyotome. Sclerotome cells migrate around the notochord and the neural tube, subsequently segregating into two distinct regions: a cranial domain comprising loosely arranged cells and a caudal region characterized by densely packed cells. The process ultimately leads to development of the vertebral bodies, arches, and transverse and spinous processes. The notochord plays a role in establishing the embryo's longitudinal axis, determining the vertebral column orientation, and guiding the formation of the nucleus pulposus of the intervertebral discs. On the other hand, the dermomyotome gives rise to the dermis and skeletal muscles [1–4] (Fig. 1). Chondrification and ossification are the final steps in the formation of the vertebrae [5]. On the molecular level, vertebral column development depends on the proper action of several signaling pathways, including Wnt, fibroblast growth factor (FGF), Notch, Hedgehog (Hh), retinoic acid (RA), transforming growth factor β (TGF-β), and bone morphogenic protein (BMP) [6–8]. The primary function of the vertebral column is to provide structural support for the body.

Vertebral malformations (VMs) is an umbrella term describing an etiologically heterogeneous group of congenital defects that may be caused by pathogenic variants in the somitogenesis genes, environmental factors, or a combination of both [9–11]. The prevalence of VMs is approximately 1–2 per 2000 live births, however, their actual incidence may be higher due to missed or delayed diagnosis [12, 13]. Depending on which process of the vertebral development has failed, VMs have been divided into segmentation, formation, mixed (both segmentation and formation), or other defects [14]. In addition to vertebral defects, fused or missing ribs or their malalignment are often noted [15]. Vertebral defects may be isolated or associated with other congenital anomalies, including congenital kyphosis or scoliosis, VACTERL association, or syndromes such as Klippel–Feil, spondylocostal dysostosis, spondylothoracic dystrophy, Alagille, Gorlin, CHARGE, Jarcho-Levin, Goldenhar or Joubert syndromes [10, 13, 16, 17]. Patients affected by VMs may be either asymptomatic or present with significant disabilities, resulting in body deformations, motor impairment, respiratory distress or chronic pain which seriously reduces their quality of life [10, 18]. Since there is no cure for VMs, treatment focuses on symptoms managed with either lifestyle or surgical interventions. Surgery is indicated mainly in younger patients with thoracolumbar anomalies and particular VMs, i.e., Klippel–Feil syndrome and congenital scoliosis [19–21]. The surgical intervention options encompass convex hemiepiphysiodesis, instrumented fusion, osteotomies, vertebrectomies, and utilization of growth-promoting systems [22].

Herein, we present a comprehensive clinical description of rare congenital vertebral column defects, provide an overview of the most relevant and recent findings concerning the molecular and environmental etiology of VMs, and discuss future research directions. In 2009 and 2013, Giampietro et al. released their two review articles in this field, and since then no other comprehensive reviews of the current literature have been published [11, 13]. Our paper attempts to fill the knowledge gap by synthesizing and interpreting the latest literature to offer new insights into the molecular background of VMs.

## Classification of VMs

**Fig. 2:**
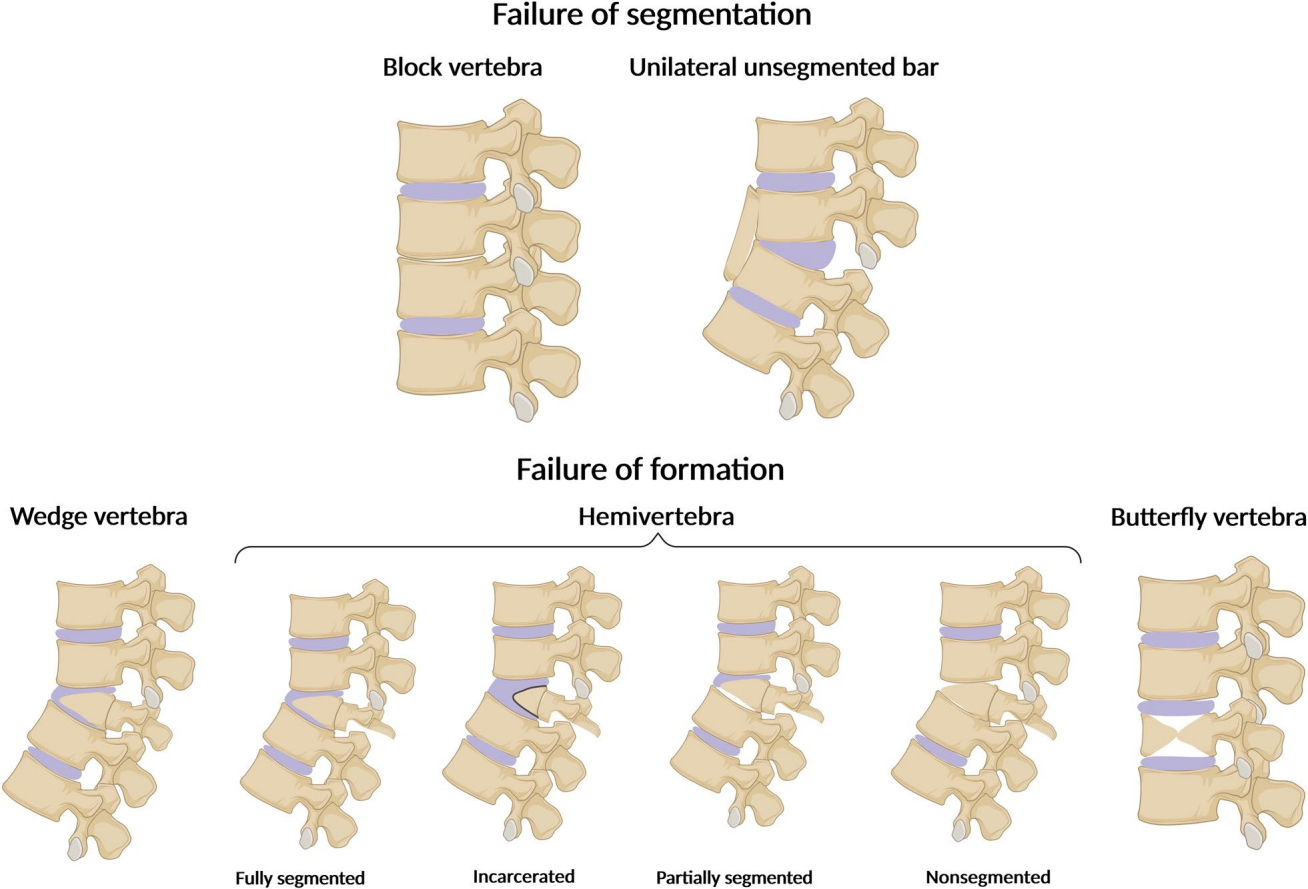
Classification of vertebrae malformations based on the segmentation or formation failures. Segmentation defects encompass block vertebra and unilateral unsegmented bar, whereas formation defects include wedge vertebra, hemivertebra, and butterfly vertebra. Hemivertebra is classified into fully segmented, incarcerated, semisegmented, and nonsegmented. Segmentation defects were illustrated using the example of the lumbar spine segment. Created with Biorender.com

Vertebral anomalies result from formation, segmentation, or simultaneous formation and segmentation defects [14]. Formation failure is due to the absence of vertebral elements occurring in the anterior, anterolateral, posterior, posterolateral, or lateral region and may be complete (hemivertebra, butterfly vertebra, vertebral aplasia) or partial (wedge vertebra). On the other hand, segmentation failure (unilateral unsegmented bar, block vertebra) arises from abnormal embryological segmentation of the vertebral column (Fig. 2).

Hemivertebra (HV) is one of the most common vertebral anomalies, with an estimated incidence from 1 to 10 per 10,000 live births, and it is mainly detected within the thoracic (Th8) and lumbar spine [23–25]. HV occurs when half of the vertebral body fails to develop (unilateral defect), and one pedicle is missing [14]. It has been shown that HV is not a supernumerary vertebra but rather an underdeveloped innate vertebra that originates from asynchronous growth of the hemimetameric pair [26]. Based on the growth pattern and positioning of the HV, the deformity is classified into four subtypes – fully segmented, incarcerated, semi-segmented, and nonsegmented [27]. Importantly, HV represents a common cause of congenital scoliosis [28]. Butterfly vertebra (BV), also termed sagittal cleft vertebra, anterior rachischisis, somatoschisis, or anterior spina bifida, is a rare vertebral malformation of unknown incidence. Due to a lack of midline fusion of two lateral chondrification centers, BV is characterized by two hemivertebrae separated by a cartilaginous septum giving the butterfly appearance on X-ray imaging [29, 30]. The defect occurs primarily in the lumbar spine or less frequently in the thoracic region, and may cause scoliosis or kyphosis [31]. Total aplasia of the vertebral body was proposed to be the consequence of chondrification center defect, and it usually leads to kyphosis. In addition, the presence of the butterfly malformation is associated with various medical conditions, such as Alagille syndrome, Crouzon syndrome, Jarcho-Levin syndrome, and Pfeiffer syndrome [32–35]. Finally, a wedge vertebra results from a unilateral asymmetry of the vertebral body where two pedicles are present. The anomaly is generally characterized by partial, unilateral chondrification and ossification [14]. Recent findings underscore the role of wedge-shaped vertebrae as a risk factor in the pathogenesis of symptomatic upper lumbar disc herniation [36].

Segmentation failure is usually observed in the cervical and lumbar spine [37]. The most frequent segmentation defect is the unilateral unsegmented bar resulting from a malformation of two or more adjacent vertebrae, leading to the fusion of over three vertebrae. The malformation results in a bony block that involves the disc spaces and facet joints, accompanied by rib fusions on the same side as the bar. A characteristic feature of an unsegmented bar is a lack of growth plates. However, the unaffected side of the vertebral column continues to grow, leading to significant spinal deformities such as congenital scoliosis [21]. The unsegmented bars can occur together with hemivertebrae, which carries a greater risk for the progression of vertebral deformation than each of these defects alone. Block vertebrae are formed due to somite segmentation failure, culminating in partial or complete fusion of the adjacent vertebrae. The morphological features of the condition include a biconcave shape at the fusion site and the presence of residual intervertebral disk material (chorda remnants) in the proximity of the fusion area. Predominantly only two vertebrae within the cervical, thoracic, or lumbar regions of the spine are affected [14]. The most frequent location for the block vertebrae is C2-C3, exhibiting a strong association with Klippel–Feil syndrome [38, 39].

## VM genetic etiology

The genetic etiology of VMs remains unexplored in the majority of affected patients. Vertebral defects may accompany the features of various, often rare, congenital syndromes. Based on the Human Phenotype Ontology database, we have listed syndromes characterized by vertebral defects, in which genetic background has been revealed (Table 1). The *KIAA1217* gene has not been associated with any syndrome yet. However, very recent investigations suggest its potential involvement in VMs. Rare variants within this gene have been identified in 10 patients with vertebral fusions and other osseous spine abnormalities [40]. In the following chapters of this review, we describe vertebral defects specific to particular segments of the spine currently intensively investigated for their genetic background. *Congenital osseous torticollis* in the form of *Klippel–Feil syndrome* was detailed as a cervical spine defect, *congenital scoliosis,* and *spondylocostal dysostoses* were depicted as thoracic/lumbar spine defects, *developmental spinal stenosis* was listed as lumbar spine defect, whereas *sacral agenesis* as a sacral spine defect. The comprehensive overview of all the genes from our publication is presented in Table 2. Our analysis shows the participation of VM genes in multiple signaling pathways, particularly in Wnt (Wnt/β-catenin, Wnt/PCP), ERK/MAPK, TGF-β, Notch, Hedgehog, BMP, and PI3K/Akt.

**Table 1:** Genes associated with pathogenesis of some VMs syndromes [40, 155–181, 224]. C–cervical, Th–thoracic, L–lumbar, SD–skeletal deformities, N/A–not applicable, ND–not determined, VBs–vertebral bodies, VMs–vertebral malformations

Gene	MIM	Syndrome	Type of vertebral defect	Others defects
*ACVR1*	102576	Fibrodysplasia ossificans progressiva	C VMs	SD (short thumbs, fifth finger clinodactyly, short broad femoral necks), deafness, mild mental retardation
*AFF4*	604417	CHOPS syndrome	C VMs (ND)	Cardiac defects (VSD, patent ductus arteriosus), intellectual disability, chronic lung disease, obesity, brachydactyly, horseshoe kidney, dysmorphic facial features, tracheomalacia, subglottic and tracheal stenosis, cryptorchidism, hearing loss
*ARSL*	300180	Chondrodysplasia punctata, X-linked recessive	Platyspondyly	Craniofacial anomalies, brachycephaly, foot syndactyly, limbs abnormalities
*COL11A1*	120280	Fibrochondrogenesis 1, Marshall syndrome, Stickler syndrome, type II	Platyspondyly	Flat midface with a small nose and anteverted nares, shortening of limb segments
*COL2A1*	120140	Kniest dysplasia	Platyspondyly	Coronal clefts, slight shortening of the ribs, dumbbell-shaped femurs
*DDRGK1*	616177	Spondyloepimetaphyseal dysplasia, Shohat type	Platyspondyly, hypoplasia of L vertebrae, square vertebrae	SD (long bone changes, short neck, L lordosis, limb shortening), hyperlaxity of joints
*EBP*	300205	Chondrodysplasia punctata, X-linked dominant	Hemivertebrae	SD (asymmetric rhizomelia, epiphyseal stippling),cataracts
*FN1*	135600	Spondylometaphyseal dysplasia, corner fracture type	Asymmetric vertebral pedicles, hypoplasia of Th12, ovoid VBs, irregular vertebrae	SD (thoracolumbar scoliosis, metaphyseal dysplasia, short stature)
*GDF11*	603936	Vertebral hypersegmentation and orofacial anomalies	C, Th, L vertebrae hypersegmentation	SD (rib abnormalities, hypermobile joints, winged scapulae), orofacial anomalies, ear anomalies
*GPC3*	300037	Simpson-Golabi-Behmel syndrome, type 1	Th hemivertebrae	Sprengel’s deformity
*GPC4*	300168	Keipert syndrome	VMs (ND)	Ribs, sternum, pelvis abnormalities
*HSPG2*	142461	Dyssegmental dysplasia, Silverman-Handmaker type	Anisospondyly	Neonatal short-limbed dwarfism
*INPPL1*	600829	Opsismodysplasia	Platyspondyly	SD (short hands/feet, short long bones, bony under mineralization, short and square metacarpals and phalanges, L kyphosis, narrow chest, small and cupped pubic bones), Eye defects (hypertelorism, proptosis/shallow orbits)
*JAG1*	601920	Alagille syndrome 1	Butterfly vertebra, Decrease in interpediculate distance in the lumbar spine	Eye defects (posterior embryotoxon and retinal pigmentary changes), heart defects (pulmonic valvular stenosis, peripheral arterial stenosis), nervous system abnormalities, facial dysmorphism (broad forehead, pointed mandible and bulbous tip of the nose and in the fingers, varying degrees of foreshortening)
*KIAA0586*	610178	Short-rib thoracic dysplasia 14 with polydactyly	Th6 butterfly vertebra	SD (small chest with short ribs, bilateral hand post-axial polydactyly, short limbs), cleft palate, lower gingiva clefts, vision defects (papillary coloboma and atrophy of the choroid-retinal inferopapillary)
*KIAA1217*	617367	N/A	C, Th fusion, hemivertebrae, wedged-shape vertebrae	SD (Sprengel deformity), cardiac defects (ASD, VSD, dextrocardia, myocarditis), central nervous system abnormalities (hydrocephalus, macrocephaly, tethered cord, cerebellar tonsillar prolapse into spinal canal, basilar invagination)
*LBR*	600024	Rhizomelic skeletal dysplasia with Pelger-Huet anomaly	Platyspondyly and ovoid VBs	SD (short limbs, shortened ribs)
*NADSYN1*	608285	Vertebral, cardiac, renal, and limb defects syndrome 3	Butterfly vertebra, hemivertebra, L, Th wedge-shaped vertebra	Rib abnormalities, heart defects (mitral insufficiency, bicuspid aortic valve, mitral valve prolapse), renal aplasia, diastematomyelia, tethered cords, hepatic polycysts
*NOTCH2*	600275	Hajdu-Cheney Syndrome	Increased anterior height of the L VBs with reduced intervertebral distances	SD (wormian bones, serpentine fibulae, bathrocephaly, irregular tooth positioning, abnormal curvature of the C spine), polycystic kidneys, ventricular septal defect, facial dysmorphism (a thin upper lip, downturned mouth, wide nasal tip, long and flat philtrum, dysplastic and posteriorly rotated ears, and short neck), hearing loss, hypothyroidism
*NSDHL*	300275	CHILD syndrome	VMs (ND)	Absence of several facial muscles, shortening of right leg, VSD
*PDE4D*	600129	Acrodysostosis 2, with or without hormone resistance	L stenosis (absence of normal interpedicular widening in the lumbar vertebrae)	SD (short stature, small hands, midface hypoplasia), developmental disability
*POGZ*	614787	White-Sutton syndrome	Hypoplasia of the C VBs	Short stature, microcephaly, non-ocular visual impairment, failure to thrive, diaphragmatic hernia, a duplicated renal collecting system
*SLC26A2*	606718	Achondrogenesis Ib Atelosteogenesis, type II De la Chapelle dysplasia	Deficient ossification in the L vertebrae, C kyphosis, scoliosis, and lumbar hyperlordosis with horizontal sacrum, flattened vertebrae with coronal clefts	SD (shortened limbs, small chest, clubfoot), respiratory insufficiency
*SLC29A3*	602782	H Syndrome	“Sandwich” vertebrae and platyspondyly	Anemia, bilateral femoral fractures
*SLC35D1*	610804	Schneckenbecken dysplasia	Retardation of the VBs ossification	SD (handle bar clavicle, bell shaped thorax, ossification of the posterior arch, interpediculate distance narrowing, sacral, pubic, tarsal ossification)
*SOX9*	608160	Campomelic dysplasia	Hypoplastic pedicles of Th vertebrae	SD (very small scapulas, dislocated hips, talipes equinovarus deformities, small thoracic cage), respiratory distress, renal and heart malformations
*SUMF1*	607939	Multiple sulfatase deficiency	VMs (ND)	Bilateral cataracts, retinal atrophy, ichthyosis, hepatosplenomegaly, psychomotor retardation
*TNFRSF11A*	602080	Paget disease of bone 2, early-onset	“Sandwich” vertebra	Osteoporosis
*TRPV4*	605427	Spondylometaphyseal dysplasia	Platyspondyly, dense wafer vertebrae	SD (congenital scoliosis, rib abnormalities, flared iliac wings, halberd pelvis, irregular proximal femoral growth plate, brachydactyly, carpal ossification delay), contracture

**Table 2:** Characterization of gene variants associated with vertebral malformations. Bial–biallelic, Comp het–compound heterozygous, Hemi–hemizygous, Het–heterozygous, Hom–homozygous, MF–multifactorial, ND–not determined; ^a^genes associated with several syndromes

Gene symbol	Zygosity	Inheritance	Bone developmental signaling pathway	References
**KFS**				
(a) Mendelian genes				
* GDF3*	Het	Mendelian	Regulator of BMP and TGFβ signaling pathways	[182, 183]
* GDF6*	Het	Mendelian	Regulator of BMP and TGFβ signaling pathways	[182, 184]
* MEOX1*	Hom, Comp het	Mendelian	Induced by TGFβ	[54, 185]
* MYO18B*	Hom, Comp het	Mendelian	Involved in PI3K/AKT/mTOR and ERK/MAPK signaling pathways	[57, 186]
(b) Candidate genes				
* BAZ1B*	Het	ND	Regulator of Wnt/β catenin signaling pathway	[61, 187]
* CDAN1*	ND	ND	Target of mTOR signaling pathway	[62, 188]
* CHRNG*	ND	ND	None	[62]
* COL6A1*	ND	ND	Involved in PI3K-Akt and ERK/MAPK signaling pathways	[62, 182]
* COL6A2*	ND	ND	Involved in PI3K-Akt and ERK/MAPK signaling pathways	[62, 182]
* FLNB*	ND	ND	Involved in MAPK and SMAD signaling pathways	[62, 182]
* FREM2*	Het	ND	Involved in BMP and ERK/MAPK signaling pathways	[61, 189]
* GLI3*	ND	ND	Involved in Hedgehog and TGFβ signaling pathways	[62, 182]
* KMT2D*	Het	ND	Regulator of Wnt/β catenin signaling pathway	[61, 182]
* MYH3*	ND	ND	A possible inhibitor of TGFβ signaling pathway	[62, 190]
* PAX1*	ND	ND	Regulator of Hedgehog signaling pathway	[62, 182]
* POR*	ND	ND	Regulator of Hedgehog signaling pathway	[62, 191]
* RIPPLY2*^a^	Hom, Comp het	ND	Regulator of Notch signaling pathway	[58, 182]
* SUFU*	Het	ND	Regulator of Hedgehog, Wnt/β catenin and Notch signaling pathways	[61, 182]
* TNXB*	ND	ND	Involved in PI3K-Akt signaling pathway	[62, 182]
* VANGL1*^a^	Het	ND	Involved in Wnt/PCP signaling pathway	[61, 182]
**CS**				
(a) Risk genes				
* TBX6*^a^	Bial, Het	MF	Regulator of Notch signaling pathway	[68, 182]
(b) Candidate genes				
Human studies				
* FBN1*	Het	ND	Involved in TGFβ and ERK/MAPK signaling pathways	[78, 182]
* PTK7*	Het	ND	Involved in Wnt/PCP and ERK/MAPK signaling pathways	[79, 182]
* SOX9*	Het	ND	Regulator of Wnt/β catenin signaling pathway, involved in BMP and FGFR3 signaling pathways	[80, 182]
* TBXT*^a^	Het	ND	Target of Wnt/β catenin signaling pathway	[76, 182]
Genes within CNVs				
* DHX40*	ND	ND	None	[75]
* DSCAM*	ND	ND	A possible regulator of ERK/MAPK signaling pathway	[75, 192]
* MYSM1*	ND	ND	Regulator of PI3K/AKT signaling pathway	[75, 193]
* NBPF20*	ND	ND	None	[75]
* NOTCH2*	ND	ND	Receptor of Notch signaling pathway, involved in NF-κB signaling pathway	[75, 182]
* RASA2*	ND	ND	Involved in G-protein, and Ras/MAPK signaling pathways	[75, 182]
* SNTG1*	ND	ND	None	[75]
Genes within DMRs				
* COL5A1*	ND	ND	Involved in PI3K/AKT/mTOR and ERK/MAPK signaling pathways	[145, 182]
* GRID1*	ND	ND	None	[145]
* GSE1*	ND	ND	None	[145]
* IGHG1*	ND	ND	Regulator of TGFβ/SMAD3 signaling pathway	[145, 194]
* IGHG3*	ND	ND	None	[145]
* IGHM*	ND	ND	None	[145]
* KAT6B*	ND	ND	A possibly regulator of Wnt/β catenin signaling pathway	[144]
* RGS3*	ND	ND	Regulator of G-protein signaling pathway, and have a function in Wnt signaling pathway	[145, 182]
* RNF213*	ND	ND	Involved in non-canonical Wnt signaling pathway	[145, 182]
* ROBO2*	ND	ND	Regulator of ERK/MAPK signaling pathway	[145, 195]
* SORCS2*	ND	ND	Regulator of Wnt/PCP signaling pathway	[145, 196]
* TNS3*	ND	ND	Regulator of membrane receptor signaling pathways	[143]
Animal studies				
* Dstyk*	Het	ND	Regulator of mTORC1/TFEB signaling pathway	[81]
**SCD**				
(a) Mendelian genes				
* DLL3*	Hom, Comp het	Mendelian	Ligand of Notch signaling pathway, involved in Wnt and Hedgehog signaling pathways	[89, 182]
* HES7*	Het	Mendelian	Target of Notch signaling pathway	[182, 197]
* LFNG*	Hom, Comp het	Mendelian	Target of Notch signaling pathway, involved in Wnt and Hedgehog signaling pathways	[91, 182]
* MESP2*	Hom, Comp het	Mendelian	Involved in Notch and FGF signaling pathways	[182, 198]
* RIPPLY2*^a^	Het	Mendelian	Regulator of Notch signaling pathway	[90, 182]
* TBX6*^a^	Het	Mendelian	Regulator of Notch signaling pathway	[69, 182]
(b) Candidate genes				
* DMRT2*	Hom	ND	Regulator of* SOX9*	[93, 199]
**DSS**				
Candidate genes				
* COX2*	ND	ND	Regulator of TGFβ signaling pathway	[99, 200]
* DCC*	ND	ND	None	[99]
* LRP5*	ND	ND	Receptor of Wnt/β catenin signaling pathway	[99, 182]
* VDR*	ND	ND	Involved in BMP and retinoic acid signaling pathways	[99, 182]
* ZNF704*	ND	ND	None	[99]
**Currarino syndrome**				
(a) Mendelian genes				
* MNX1*	Het	Mendelian	Regulator of PI3K/AKT/mTOR and Wnt/β catenin signaling pathways	[107, 201, 202]
(b) Candidate genes				
* ARID5A*	ND	ND	NF-κB signaling pathway activates *ARID5A* expression	[106, 203]
* CDH2*	ND	ND	Involved in Wnt/β catenin signaling pathway	[106, 182]
* ETV3L*	ND	ND	Regulator of FGF signaling pathway	[106, 204]
* HOXB4*	ND	ND	Regulator of Wnt/β catenin signaling pathway	[106, 205]
* ITIH2*	ND	ND	None	[106]
* NCAPD3*	ND	ND	Involved in NF-κB signaling pathway	[106, 206]
* TLE4*	ND	ND	Regulator of canonical Wnt, Notch and TGFβ signaling pathways	[106, 182]
**NTDs**				
Risk genes				
* CCL2*	Het, Hom	MF	Regulator of PI3K-AKT and ERK/MAPK signaling pathways	[110, 207]
* FUZ*	Het	MF	Involved in Hedgehog signaling pathway	[113, 182]
* VANGL1*^a^	Het	MF	Involved in Wnt/PCP signaling pathway	[112, 182]
* VANGL2*	Het	MF	Involved in Wnt/β catenin signaling pathway	[109, 182]
* TBXT*^a^	Het	MF	Target of Wnt/β catenin signaling pathway	[111, 182]
* AMOT*	ND	MF	Involved in Hippo-Merlin signaling pathway	[122, 182]
* ARHGAP36*	ND	MF	Regulator of Hedgehog signaling pathway	[122, 208]
* CELSR1*	ND	MF	Involved in Wnt/PCK signaling pathway	[124, 209]
* COL15A1*	ND	MF	Involved in ERK signaling pathway	[122, 182]
* DACT1*	ND	MF	Involved in Wnt signaling pathway	[126, 182]
* DISP2*	ND	MF	Involved in Hedgehog signaling pathway	[125, 182]
* DLC1*	ND	MF	Involved in MAPK signaling pathway	[120, 210]
* DTX1*	ND	MF	Regulator of Notch signaling pathway	[122, 182]
* FREM2*^a^	ND	MF	Involved in BMP and ERK/MAPK signaling pathways	[125, 188]
* FZD6*	ND	MF	Receptor of Wnt/β catenin signaling pathway	[125, 182]
* GPR50*	ND	MF	Regulator of Notch signaling pathway	[122, 211]
* GRHL3*	Het, Hom	MF	None	[119]
* ITGB1*	ND	MF	Involved in PI3K/Akt signaling pathway	[120, 212]
* MTHFR*	ND	MF	None	[120]
* MYO1E*	ND	MF	Involved in ERK signaling pathway	[120, 182]
* NKRF*	ND	MF	Regulator of NF-κB signaling pathway	[122, 182]
* PAX3*	Het	MF	Involved in Wnt, Hedgehog and Notch signaling pathways	[123, 182]
* PRICKLE1*	ND	MF	Regulator of Wnt/β catenin signaling pathway	[125, 182]
* PTK7*^a^	Het	MF	Involved in Wnt/PCP and ERK/MAPK signaling pathways	[118, 182]
* RXRγ*	ND	MF	Involved in retinoic acid signaling pathway	[122, 182]
* SCRIB*	Het	MF	Involved in MAPK signaling pathway	[121, 124, 182]
* SHROOM3*	ND	MF	None	[123]
* TKTL1*	ND	MF	None	[122]
**CDS**				
Risk genes				
* CELSR1*^a^	Het	MF	Involved in Wnt/PCK signaling pathway	[141, 209]
* VANGL1*^a^	Het	MF	Involved in Wnt/PCP signaling pathway	[112, 182]
**IDs**				
Genes within DMRs				
* CDKN1C*	Het	Mendelian	None	[150, 151]
* H19*	ND	ND	Involved in canonical Wnt signaling pathway	[150, 151, 182]
* IGF2*	Het	Mendelian	Involved in IGF2 signaling pathway	[150, 151, 182]
* KCNQ1OT1*	ND	ND	Regulator of BMP signaling pathway	[151, 182]
**Other genes (**Table 1**)**				
Mendelian genes				
* ACVR1*	Het	Mendelian	Regulator of BMP, TGF-β, Akt and NF-κB signaling pathways	[155, 182]
* AFF4*	Het	Mendelian	A possible regulator of BMP signaling pathway	[156, 213]
* ARSL*	Hom	Mendelian	None	[157]
* COL11A1*	Het	Mendelian	Involved in ERK/MAPK and PI3K/AKT/mTOR signaling pathways	[158, 182]
* COL2A1*	Het	Mendelian	Involved in PI3K/AKT/mTOR and ERK/MAPK signaling pathways	[159, 182]
* DDRGK1*	Hom, Comp het	Mendelian	Regulator of NF-κB signaling pathway	[160, 214]
* EBP*	Het	Mendelian	None	[161]
* FN1*	Het	Mendelian	Involved in ERK/MAPK and PI3K/AKT/mTOR signaling pathways	[162, 182]
* GDF11*	Het	Mendelian	Involved in TGF-β signaling pathway	[163, 182]
* GPC3*	Hemi	Mendelian	Regulator of Wnt, Hedgehog, FGF and BMP signaling pathways	[164, 215]
* GPC4*	Hemi	Mendelian	Involved in Wnt/PCP signaling	[165, 182]
* HSPG2*	Hom, Comp het	Mendelian	Involved in ERK signaling pathway	[166, 182]
* INPPL1*	Hom, Comp het	Mendelian	Regulator of PI3K-Akt and NF-κB signaling pathways	[167, 182]
* JAG1*	Het	Mendelian	Regulator of Notch signaling pathway	[168, 182]
* KIAA0586*	Hom, Comp het	Mendelian	Involved in Hedgehog signaling pathway	[169, 182]
* LBR*	Hom, Het, Comp het	Mendelian	None	[170]
* NADSYN1*	Het	Mendelian	None	[171]
* NOTCH2*^a^	Het	Mendelian	Receptor of Notch signaling pathway, involved in NF-κB signaling pathway	[172, 182]
* NSDHL*	Het	Mendelian	Regulator of TGF-β and Hedgehog signaling pathways	[173, 216]
* PDE4D*	Het	Mendelian	Involved in cAMP signaling pathway	[174, 182]
* POGZ*	Het	Mendelian	A possible regulator of Wnt signaling pathway	[175, 217]
* SLC26A2*	Hom, Comp het	Mendelian	Regulator of FGFR3 signaling pathway in mouse models	[176, 218]
* SLC29A3*	Hom, Comp het	Mendelian	Regulator of insulin signaling pathway	[177, 219]
* SLC35D1*	Hom, Comp het	Mendelian	Candidate gene for Notch signaling pathway	[178, 220]
* SOX9*^a^	Het, Hom	Mendelian	Regulator of Wnt/β catenin signaling pathway, involved in BMP and FGFR3 signaling pathways	[182, 224]
* SUMF1*	Hom, Comp het	Mendelian	Regulator of FGF signaling pathway	[179, 221]
* TNFRSF11A*	Het	Mendelian	Involved in PI3K-Akt and NF-kappaB signaling pathways	[180, 182]
* TRPV4*	Het	Mendelian	Regulator of TGF-β signaling pathway	[181, 222]
(b) Candidate genes				
* KIAA1217*	Het	Mendelian	Regulator of Notch and Wnt/β-catenin signaling pathways	[40, 223]

### Cervical spine

#### Congenital osseous torticollis—Klippel–Feil syndrome

**Fig. 3:**
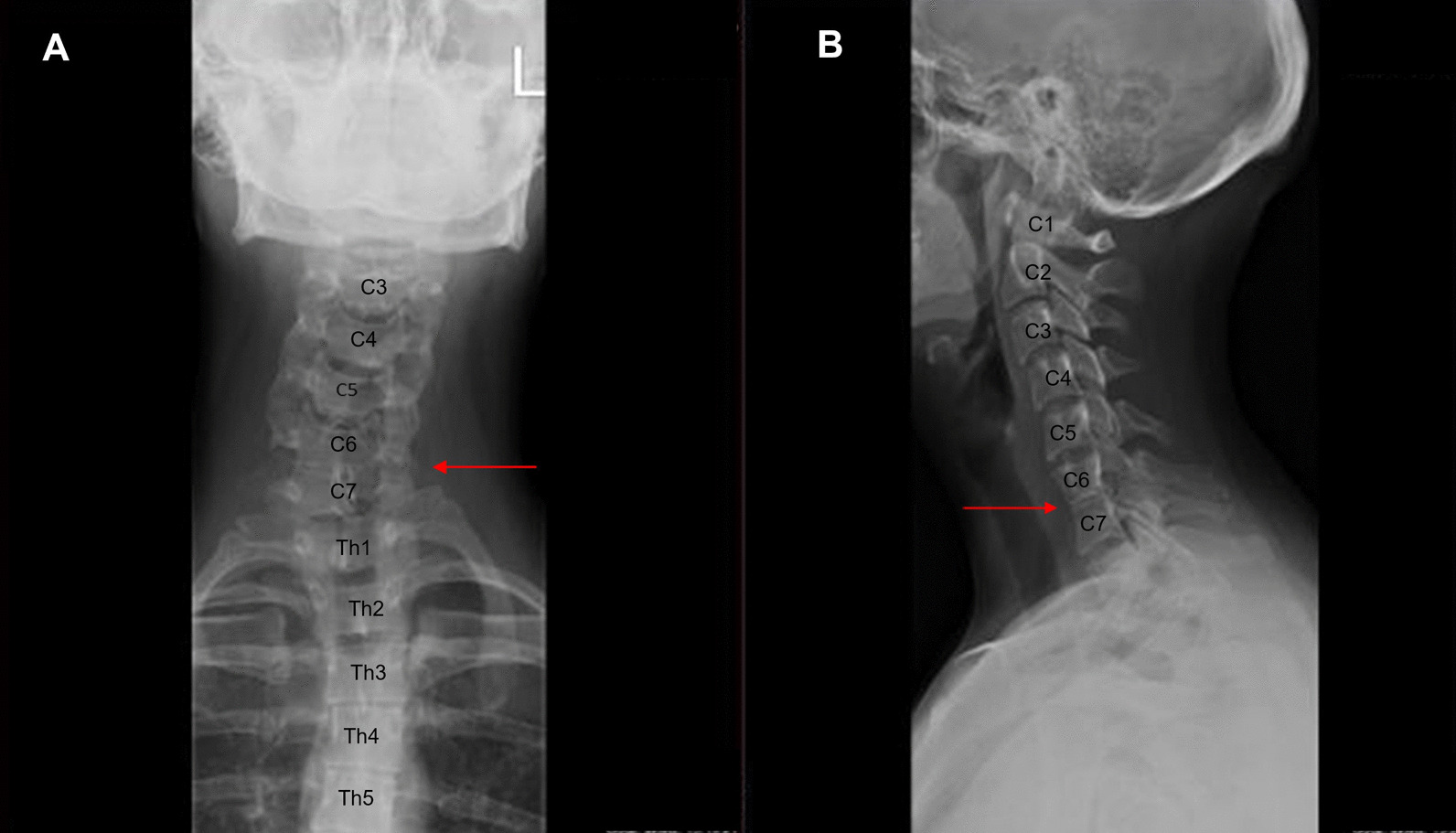
Anteroposterior (**A**) and lateral (**B**) cervical spine radiographs showing vertebrae fusion at C6-C7 in a patient with Klippel–Feil syndrome

Klippel–Feil syndrome (KFS) is a complex skeletal disorder characterized by the fusion of at least two cervical vertebrae, initially reported by Maurice Klippel and Andre Feil [41]. Congenital vertebral fusions may occur at any cervical spine level, although the most often affected vertebrae are C2-C3 and C5-C6 [42]. Since the first description of this syndrome, three morphological subtypes of the disorder have been identified: type I, characterized by the fusion of cervical and upper thoracic vertebrae, type II, with only one or two pairs of fused cervical vertebrae (Fig. 3), and type III, with the fusion of cervical vertebrae combined with the fusion of lower thoracic or lumbar vertebrae [43]. KFS is reported in 1 of 40,000 to 42,000 newborns worldwide. However, the incidence of this syndrome remains underreported due to a lack of population screening studies and frequent asymptomatic occurrence. Studies involving 2917 patients at the emergency department and 131 patients with cervical spondylotic myelopathy, who underwent spine imaging, revealed the prevalence of KFS to be 0.58% and 3.82%, respectively [42, 44]. A diagnosis of KFS is based on the clinical triad, which includes a short neck, low-set posterior hairline, and limited head and neck movements. Notably, only 34–74% of the affected individuals manifest all three symptoms [45]. KFS can be isolated or associated with numerous abnormalities, including scoliosis, Sprengel deformity, spina bifida occulta, renal abnormalities, vision and hearing impairment, congenital heart defects, and neurological anomalies [46–48].

There are four genetic forms of KFS with dominant and recessive inheritance: KFS1, KFS2, KFS3, and KFS4 (Table 3). In KFS patients, many chromosomal abnormalities have been reported, i.e., inv(8)(q22.2q22.3); t(5;17)(q11.2;q23); inv(2)(p12q34) or t(5;8)(q35.1;p21.1) [49–52]. Furthermore, according to Online Mendelian Inheritance in Man (OMIM), pathogenic variants in different genes are associated with autosomal dominant KFS, i.e., *GDF6* (MIM: 601147), *GDF3* (MIM: 606522), and autosomal recessive KFS, i.e., *MEOX1* (MIM: 600147), and *MYO18B* (MIM: 607295). The *GDF3* and *GDF6* genes are members of the TGF-β/BMP family, and their protein products are essential for forming and developing bones and joints. The *MEOX1* gene encodes a homeobox protein MOX-1, a transcription factor expressed in somites. MOX-1 regulates separation of vertebrae from one another during early development. Despite the clinical heterogeneity of KFS, the patients harboring pathogenic variants in the *MEOX1* gene display multiple common features, i.e., Sprengel’s deformity, congenital scoliosis, and an ectopic omovertebral bone [53, 54]. The *MYO18B* gene encodes an unconventional class XVIII myosin, mainly expressed in human cardiac and skeletal muscle. The protein plays a potential role in cellular processes and transcriptional regulation of muscle-specific genes [55]. A null variant in *MYO18B* was linked to a novel developmental disorder that combines KFS and myopathy. Noteworthy, only a small subset of KFS cases could be explained by pathogenic variants in one of the four mentioned genes [56].

**Table 3:** Genetic classification of Klippel–Feil syndrome. MIM–Mendelian Inheritance in Men

Genetic form of Klippel–Feil syndrome (KFS)	Inheritance	Gene	Overlap with morphological types of Klippel–Feil syndrome	References
KFS1	Autosomal dominant	*GDF6* (MIM: 601147)	Types I, II, and III	[184]
KFS2	Autosomal recessive	*MEOX1* (MIM: 600147)	Types I, II, and III	[54]
KFS3	Autosomal dominant	*GDF3* (MIM: 606522)	Type II	[183]
KFS4	Autosomal recessive	*MYO18B* (MIM: 607295)	None	[57]

Multiple genes have been proposed as potential candidates responsible for KFS. A homozygous frameshift variant in *RIPPLY2* was identified in a patient suffering from KFS with heterotaxy. Studies indicated that variants in *RIPPLY2* could be responsible for a new type of KFS. However, further research is required to verify this possible link [57, 58]. Mouse models also identified some variants in the PAX gene family and the Notch signaling pathway as potential genetic cause of the described disorder [59]. Abnormalities in *PAX1* have been identified in 8 out of 63 patients with KFS [60]. Furthermore, researchers found out that among five new candidate genes (*BAZ1B*, *FREM2*, *VANGL1*, *SUFU*, and *KMT2D*), the variants in *BAZ1B* had the strongest association with KFS [61]. On the other hand, a study by Li et al. revealed 11 pathogenic missense variants in eight KFS patients, including *COL6A1*, *COL6A2*, *CDAN1*, *CHRNG*, *FLNB*, *GLI3*, *MYH3*, *POR*, and *TNXB*, but none within KFS-related genes – *GDF6*, *GDF3*, *MEOX1*, and *MYO18B* [62].

### Thoracic/lumbar spine

#### Congenital scoliosis

Congenital scoliosis (CS) is a spinal deformity resulting from the abnormal shape of vertebrae (hemivertebrae, butterfly vertebrae, wedge vertebrae), segmentation failure, or a combination of both [63, 64]. Hemivertebrae are the most common cause of CS. Many CS patients also have defects in other organs, particularly in the heart and the genitourinary system [65]. This condition is estimated to occur in 1 per 2000 live births and manifests as a lateral curvature of the spine (Cobb angle) exceeding 10 degrees. The indication for CS surgery depends on the degree of CS at the time of diagnosis and the disease progression.

The genetic basis of CS is only partially explained. Approximately 10% of the patients harbor heterozygous *TBX6* loss-of-function variants or a deletion copy-number variant (CNV) within chromosome 16p11.2, including the *TBX6* gene [66–68]. Wu et al. reported that CS patients with *TBX6* loss-of-function variants carry an additional hypomorphic variant on the second *TBX6* allele, which is a specific haplotype corresponding to one of the following common SNVs: *rs2289292*, *rs3809624*, and *rs3809627* [68]. In two subsequent studies, researchers found these variations in *TBX6* in about 9.6% and 7.14% of CS patients, respectively [69, 70]. *TBX6* belongs to the T-box family and encodes a transcription factor controlling presomitic mesoderm segmentation and differentiation during development [71, 72]. In 2019, Liu et al. defined *TBX6*-associated congenital scoliosis (TACS) as a unique clinically recognizable subtype of CS [73, 74].

In addition to 16p11.2 deletion, involving the *TBX6* gene, a recent study revealed novel CNVs carried by CS individuals [75]. Lai et al. identified recurrent CNVs encompassing three scoliosis-related genes, including *NOTCH2, DSCAM*, and *SNTG1* and four genes (*DHX40*, *NBPF20*, *RASA2*, and *MYSM1*) possibly linked to skeletal abnormalities [75].

New CS candidate genes have also been proposed, i.e., *TBXT*, *FBN1*, *PTK7*, *SOX9*, and *Dstyk* [76–81]. Similarly to *TBX6*, *TBXT* (also known as Brachyury or T), a member of the T‐box family, is highly expressed in the notochord and is involved in mesoderm formation and axial elongation [82]. According to some studies, *FBN1* may trigger CS by upregulating TGF-β signaling, which is essential for skeletal development [78, 83]. The third candidate gene, *PTK7*, plays a crucial role in canonical and non-canonical Wnt signaling, whereas the fourth CS candidate gene, *SOX9*, is involved in chondrocyte differentiation, notochord maintenance, and demarcation of intervertebral disc compartments [84–86]. Finally, variants of *Dstyk* may result in CS-like VMs in zebrafish due to disrupting the formation of the notochord vacuole through the mTORC1/TFEB pathway [81].

#### Spondylocostal dysostosis

Another congenital spinal disorder, spondylocostal dysostosis (SCD), shares a similar phenotype with CS. SCD is a rare genetic defect characterized by malformations of the ribs and vertebrae (hemivertebrae, butterfly vertebrae, fusion, block, or mixed abnormalities). SCD patients often present with a short neck, short trunk, and scoliosis [17, 87]. To date, SCD has been classified into seven subtypes based on their phenotypes and disease genes: SCD1 with pathogenic variants in *DLL3*, *SCD2* with pathogenic variants in *MESP2*, *SCD3* with pathogenic variants in *LFNG*, *SCD4* with pathogenic variants in *HES7*, *SCD5* with pathogenic variants in *TBX6*, SCD6 with pathogenic variants in *RIPPLY2*, and SCD7 with pathogenic variants in *DLL1*. All these disorders are inherited in an autosomal recessive manner. However, SCD5, in addition to autosomal recessive transmission may also present autosomal dominant inheritance pattern [68, 88–92]. It has been shown that SCD may co-occur with additional cervical and sacral spine malformations or costovertebral malformations. In such phenotypes, pathogenic variants are identified in *LFNG* or *DRMT2*, respectively [91, 93, 94]. The results of a functional analysis of the missense *LFNG* variant (p.Phe188Leu) showed no difference in protein expression between the mutant and wild-type mice [91]. In contrast, the *Dmrt2* knock-out mice displayed a similar phenotype to a human neonate with SCD, indicating that pathogenic variants in *DMRT2* may be related to a new subtype of SCD [93].

### Lumbar spine

#### Developmental spinal stenosis

Developmental spinal stenosis (DSS), also known as congenital lumbar spinal stenosis, is likely caused by fetal and postnatal abnormal development of the posterior spinal elements [95, 96]. The most common clinical features of DSS include a narrow spinal canal, enlarged lamina, and short pedicles [97]. In some cases, the lumbar vertebrae give the spinal canal a trefoil appearance that leads to lumbar and sacral nerve compression [98]. Genetic predisposition to DSS differs between the upper (L1-L4) and the lower (L5-S1) lumbar spine levels. Genome-Wide Association Study showed that L4 and L5 vertebrae DSS-associated SNVs were located within the *ZNF704*, and *DCC* genes, respectively. In addition, three candidate genes, i.e., *LRP5*, *COX2*, and *VDR* can contribute to DSS [99]. DSS is often associated with achondroplasia, a type of skeletal dysplasia resulting from specific *FGFR3* activating alterations. Such a complication leads to neurologic symptoms in affected individuals and thus requires surgical interventions [100–102]. Sporadically, congenital thoracolumbar stenosis is also noted in alkaptonuria, as described recently [103].

### Sacral spine

#### Sacral agenesis

Sacral agenesis is a congenital absence of the entire sacrum. The classic form of sacral agenesis is autosomal dominant Currarino syndrome (MIM: 176450), in which partial agenesis, i.e., hemisacrum, within S2-S5 vertebrae occurs. In addition, patients present with anorectal malformations, a presacral mass (anterior meningocele, enteric cyst, or presacral teratoma), and urogenital anomalies [104]. Over twenty years ago, a causative gene for this syndrome was found, i.e., *MNX1,* also known as *HLXB9* [105]. Recently, whole exome sequencing studies of 6 patients with Currarino syndrome revealed 7 variants that might be linked to the disorder, i.e., a de novo variant in *ETV3L* (p.Val126Ile), a de novo variant in *NCAPD3*, a variant in *ARID5A* (p.Arg55Leu), a missense variant in *CDH2* (p.Arg151Ser), a variant in *ITIH2* (p.Ile541Ilefs12), a variant in *HOXB4* (p.Lys16Asn), and variant in *TLE4* (p.Ser650Leu) [106, 107].

## The role of environmental factors and epigenetics in congenital spinal deformities

### The role of environmental factors

#### Neural tube defects

Neural tube defects (NTDs) represent a group of congenital anomalies characterized by incomplete neural tube closure during embryonic development. The defects result from a complex interplay of genetic and environmental factors. NTDs encompass a heterogeneous spectrum of congenital anomalies, including anencephaly, spina bifida (SB), encephalocele, and craniorachischisis [108]. Genetic factors play a key role in the etiology of NTDs, with intragenic susceptibility variants identified in multiple genes, including *CCL2* (MIM: 158105), *FUZ* (MIM: 610622), *VANGL1* (MIM: 610132), *VANGL2* (MIM: 600533), and *TBXT* (MIM: 601397) [109–113]. The pathogenic variant in the *CCL2* gene predisposes to the development of SB. Notably, the *CCL2* gene regulates the export level of monocyte chemotactic protein-1 following treatment with interleukin-1-β in vitro [114]. Research has shown that maternal hyperthermia in the first trimester of pregnancy is associated with a twofold increase in the incidence of SB [115]. Hence, inflammation and increased body temperature, mediated by chemokines, may be contributing factors in the pathogenesis of SB. Jensen et al. linked the *CCL2A*(-2518)G promoter polymorphism with SB, as the allele could attenuate the response to infection [110]. Another predisposing gene in NTDs, expressed in the emerging neural tube, is the *FUZ* gene. Seo et al. found 5 missense heterozygous pathogenic substitutions in *FUZ* in an Italian cohort, i.e., p.Pro39Ser, p.Asp354Tyr, p.Arg404Glu, p.Gly140Glu, and p.Ser142Thr. The variants disrupt primary cilia formation and affect directional cell movement, which are crucial processes in developing the spinal neural tube [113]. Furthermore, several heterozygous missense pathogenic variants within the *VANGL1* and *VANGL2* genes have been associated with a subset of human NTDs. Merello et al. suggested a correlation between three heterozygous missense variants of *VANGL1*, p.Ala187Val, p.Asp389His, and p.Arg517His, and the occurrence of NTDs [116]. Interestingly, another research group has indicated a predisposition of pathogenic variants in *VANGL2* (p.Ser84Phe, p.Arg353Cys, and p.Phe437Ser) to an increased risk of cranial NTDs in human fetuses [109]. Finally, researchers have identified a pathogenic variant in the *TBXT* gene, TIVS7-2, in individuals suffering from meningomyelocele. The variant has been concomitantly correlated with elevated predisposition to SB [117]. Numerous studies have also identified other risk-candidate genes such as *AMOT*, *ARHGAP36*, *CELSR1*, *COL15A1*, *DACT1*, *DISP2*, *DLC1*, *DTX1*, *FREM2*, *FZD6*, *GPR50*, *GRHL3*, *ITGB1*, *MTHFR*, *MYO1E*, *NKRF*, *PAX3*, *PRICKLE1*, *PTK7*, *RXRγ*, *SCRIB*, *SHROOM3*, and *TKTL1* [118–126]. Despite identifying susceptibility variants responsible for NTDs, recent studies have revealed a significant role of environmental factors in the etiology of NTDs. A prospective study has demonstrated that fever during the first month of pregnancy increases the risk of NTDs [115]. Furthermore, a systematic review and meta-analysis conducted in 2005 confirmed that hyperthermia in early pregnancy is a risk factor for NTDs [127]. Other significant factors contributing to the development of NTDs are maternal diabetes and obesity. Specifically, teratogenic implications of hyperglycemia and hyperinsulinemia increase cellular apoptosis within the developing embryonic neural plate. Women diagnosed with diabetes manifest a notable 2- to tenfold escalation in the risk of NTDs, whereas women affected by obesity demonstrate a 1.5- to 3.5-fold increase, with the severity of risk correlating with maternal body mass index [128–130]. Thirdly, inadequate maternal nutritional status during pregnancy, i.e., deficiencies in folate, zinc, and B12, is a factor in the increased risk of NTDs. Notably, research strongly supports the association between folate deficiency and NTDs [131, 132]. The recommended folic acid dosage for women with a previous NTD-complicated pregnancy is 4 mg/day [133]. Among antiepileptic drugs, valproic acid is the most widely recognized teratogenic drug associated with NTDs. The risk of NTDs related to valproate exposure appears to be dose-dependent, necessitating cautionary measures to avoid its use or to limit the dosage [134]. Finally, alcohol and caffeine consumption and maternal exposure to passive smoking are potential risk factors, however, more studies are needed [135–137].

##### Caudal dysgenesis syndrome

Caudal dysgenesis syndrome (CDS; MIM: 600145), also classified as neural tube defect, is a form of sacral agenesis, in which various heterogeneous constellations of symptoms are observed. The CDS phenotype encompasses defects of caudal derivatives, such as anomalies affecting the caudal spine, the spinal cord, the hindgut, the urogenital system, and sporadically the lower extremities (sirenomelia) [138, 139]. Amongst CDS causes, one may list maternal insulin-dependent diabetes during pregnancy (detected in 15–25% of mothers who gave birth to affected children) and pathogenic variants within the *VANGL1* or *CELSR1* genes [112, 140, 141]. Furthermore, the influence of exogenous substances on the fetus, including retinoic acid and insulin, is also a potential risk factor [142].

### The role of epigenetics

Epigenetic factors represent another potential mechanism that may be involved in the pathogenesis of VMs. The epigenetic genes involved in the etiology of vertebral defects are summarized in Table 4. Recent studies showed that aberrant DNA methylation might be linked with the pathogenesis of CS. As compared with healthy individuals, CS patients showed hypermethylation in *KAT6B*, *TNS3*, *IGHG1*, *IGHM*, *IGHG3*, *RNF213*, and *GSE1*, and hypomethylation in *SORCS2*, *COL5A1*, *GRID1*, *RGS3*, and *ROBO2* [143–145]. Moreover, DNA methylation is a critical mechanism in the process of genomic imprinting, an epigenetic mode of inheritance in which genes are expressed exclusively from one parental chromosome, depending on their parental origin. These epigenetic modifications during gametogenesis have been implicated in the etiology of several congenital imprinting disorders (IDs), which present with different clinical features. Silver–Russell syndrome (SRS) and Beckwith–Wiedemann syndrome (BWS) represent examples of imprinting disorders associated with VMs [146]. SRS is characterized by growth retardation, macrocephaly at birth, and dysmorphic facial features (triangular face, prominent forehead). Symptoms associated with VMs include scoliosis, kyphosis, kypho-lordosis, lumbar hypomobility, lumbar hypolordosis with lumbar hypomobility, and abnormally high lumbar vertebrae [147–149]. Hypomethylation at the imprinting control region 1 (ICR1) located on chromosome 11p15.5, resulting from the loss of paternal methylation, constitutes a primary cause of SRS. This epigenetic aberration affects the expression of growth-regulatory genes, i.e., *IGF2* and *H19*. Furthermore, patients with SRS carry maternal uniparental disomy of chromosomes 7, 14, 16, and 20, aberrant methylation of 14q32.2, maternal gain-of-function variants in *CDKN1C*, and paternal loss-of-function variants in *IGF2* [150]. BWS manifests clinical features, including macrosomia, macroglossia, abdominal wall defects, and elevated risk for embryonal tumors [151]. Additionally, a recent study identified painful scoliosis with lateralized overgrowth as one of the consequences of BWS [152]. Analogously to SRS, most BWS cases exhibit DNA methylation alterations at the chromosomal locus 11p15.5-11p15.4. In contrast to SRS, BWS is typified by hypermethylation at the ICR1 and hypomethylation at the ICR2, which result in dysregulation of three imprinted genes shared with SRS, namely *IGF2*, *H19*, and *CDKN1C*, and the *KCNQ1OT* gene [151].

**Table 4:** Description of epigenetic genes associated with vertebral malformations pathogenesis. BWS–Beckwith–Wiedemann syndrome, CS–Congenital scoliosis, ICR1–Imprinting control region 1, ICR2–Imprinting control region 2

Gene	Epigenetic change	Conditions	Country of the study	Year of the study	References
*CDKN1C*	Hypomethylation of the ICR2 in the imprinted region 11p15.5	BWS	The United States of America	2003	[225]
*COL5A1*	Gene hypomethylation	CS	China	2021	[145]
*GRID1*	Gene hypomethylation	CS	China	2021	[145]
*GSE1*	Gene hypermethylation	CS	China	2021	[145]
*H19* *IGF2*	Hypermethylation of the ICR1 in the imprinted region 11p15.5	BWS	United Kingdom	1997	[226]
	Hypomethylation of the ICR1 in the imprinted region 11p15.5	SRS	Switzerland	2009	[227]
*IGHG1*	Gene hypermethylation	CS	China	2021	[145]
*IGHG3*	Gene hypermethylation	CS	China	2021	[145]
*IGHM*	Gene hypermethylation	CS	China	2021	[145]
*KAT6B*	Gene hypermethylation	CS	China	2020	[144]
*KCNQ1OT*	Hypomethylation of the ICR2 in the imprinted region 11p15.5	BWS	The Netherlands	2001	[228]
*RGS3*	Gene hypomethylation	CS	China	2021	[145]
*RNF213*	Gene hypermethylation	CS	China	2021	[145]
*ROBO2*	Gene hypomethylation	CS	China	2021	[145]
*SORC2*	Gene hypomethylation	CS	China	2021	[145]
*TNS3*	Gene hypermethylation	CS	China	2022	[143]

## Future perspectives and conclusions

Studies regarding the genetic background of VMs are ongoing worldwide. However, their main limitations remain the rare occurrence of VMs, clinical heterogeneity of these defects, and the economic barrier that all impede performing large cohort research screening using advanced technologies, including whole-genome sequencing, transcriptome profiling via RNA-seq, third-generation sequencing, single-cell sequencing, and other more sophisticated functional studies.

Given the phenotypic heterogeneity of VMs, the application of exact classification systems appears critical for clinical recognition and, next, molecular background research. Studies of clinically homogenous groups of VMs patients are highly needed for identifying the causative genetic lesions underlying vertebral defects and closing the knowledge gap in this area. Simultaneously, exploring the potential contribution of epigenetic factors to the development of vertebral disorders is an interesting avenue for future research. While studies into the epigenetics of CS and IDs have yielded promising results in recent years, there is a knowledge gap in the potential role of epigenetics in other described syndromes. Recent studies on rare diseases such as chromatinopathies and Kabuki syndrome have underscored the crucial role of genome-wide DNA methylation analysis in establishing definitive molecular diagnoses, particularly in the cases where initial genetic screenings yield negative results. Simultaneously, integrating genotype, phenotype, and epigenetic factors has been proposed as a promising approach to unraveling the molecular basis of rare diseases [153, 154]. So far, only one promising study has explored the global genome-wide methylation profile in CS patients, albeit with a small sample size of n = 4 [145]. To expand the scope of methylation investigations in CS and initiate studies in other described VMs disorders, novel methods such as comprehensive whole-genome bisulfite sequencing and methylome arrays covering approximately 850,000 loci could be used. We assume that integrative analyses incorporating multi-omics data, encompassing (epi-)genomic, transcriptomic, and chromatin studies, hold significant promise in providing a comprehensive molecular picture of VMs. Furthermore, to our knowledge, there are no cis-regulatory variants in the non-coding DNA described so far in the medical literature that are causative for VMs. Thus, pathogenic variants located in the regulatory elements of the genes involved in embryonic vertebral development represent another putative disease mechanism. Such causative changes can be identified via array comparative genomic hybridization and whole-genome sequencing analyses.

Importantly, the complexity of VMs etiology cannot be excluded. The involvement of external environmental causes such as maternal drug intake, maternal diseases during pregnancy, or other yet unidentified environmental factors affecting the developing fetus or possibly parents before pregnancy, should also be considered. In VMs disorders influenced by environmental factors, the range of structural abnormalities can differ significantly based on the timing of exposure to these factors during embryonic development and the intensity of their impact. As a result, the affected individuals may display a variety of anomalies, with differences in the type and severity of malformations. Conversely, genetic disorders show a more consistent pattern of inheritance and recurrence within families.

In conclusion, the described heterogeneity of VMs highlights the need for interdisciplinary research approaches that integrate genetics, environmental factors, and epigenetic mechanisms.

## Data Availability

Not applicable.

## Abbreviations

VMs: Vertebral malformations
FGF: Fibroblast growth factor
HG: Hedgehog
RA: Retinoic acid
TGF-β: Transforming growth factor β
BMP: Bone morphogenic protein
HV: Hemivertebra
BV: Butterfly vertebra
KFS: Klippel–Feil syndrome
OMIM: Online Mendelian Inheritance in Man
CS: Congenital scoliosis
CNV: Copy-number variant
TACS: *TBX6*-Associated congenital scoliosis
SCD: Spondylocostal dysostosis
DSS: Developmental spinal stenosis
NTDs: Neural tube defects
SB: Spina bifida
CDS: Caudal dysgenesis syndrome
IDs: Imprinting disorders
SRS: Silver–Russell syndrome
BWS: Beckwith–Wiedemann syndrome
ICR1: Imprinting control region 1
ICR2: Imprinting control region 2
